# The *In-Vitro* Antitumor Effects of AST-3424 Monotherapy and Combination Therapy With Oxaliplatin or 5-Fluorouracil in Primary Liver Cancer

**DOI:** 10.3389/fonc.2022.885139

**Published:** 2022-07-22

**Authors:** Yu Zhang, Shukui Qin, Jiaojiao Chao, Yan Luo, Yandi Sun, Jianxin Duan

**Affiliations:** ^1^ Department of Graduate School, Nanjing University of Chinese Medicine, Nanjing, China; ^2^ Department of Medical Oncology Center, BaYi Affiliated Hospital, Nanjing, China; ^3^ Department of Biochemistry and Cancer Institute of the Second Affiliated Hospital (Key Laboratory of Cancer Prevention and Intervention of China National Ministry of Education (MOE)), Zhejiang University School of Medicine, Hangzhou, China; ^4^ Ascentawits Pharmaceuticals, Ltd., Shenzhen, China

**Keywords:** AST-3424, AKR1C3, liver cancer, chemotherapy, synergy

## Abstract

**Background:**

Primary liver cancer (PLC) is a common and highly lethal malignancy in the world. Approximately 85% of PLC is hepatocellular carcinoma (HCC), and this study mainly focuses on HCC. The onset of liver cancer is insidious and often complicated with basic liver disease. Meanwhile, its clinical symptoms are atypical, and the degree of malignancy is high. What is worse is that its treatment is difficult, and the prognosis is poor. All these factors make its mortality close to its incidence. AST-3424 is a prodrug of a potent nitrogen mustard, which targets the tumor by its specific and selective mode of activation and results in the concentration of the drug in the tumor and plays a higher intensity of antitumor effect with reduced side effects. The purpose of this study was to explore the *in-vitro* antitumor activity and mechanism of AST-3424 monotherapy and combination therapy with oxaliplatin (OXA) or 5-fluorouracil (5-Fu). Moreover, it can provide an experimental basis for further studies.

**Methods:**

Tumor growth of HCC cells was examined by using the Cell Counting Kit-8 (CCK-8), flow cytometry, and clone formation assays. Tumor migration of HCC cells was examined by using the Transwell assay. The *in-vitro* antitumor activity of AST-3424 monotherapy and combination therapy with OXA and 5-Fu was quantified by growth and metastasis inhibition rate. The underlying molecular mechanism was investigated by using Western blotting.

**Results:**

The inhibiting effects of AST-3424 were significant in both HepG2 cells and PLC/PRF/5 cells. Moreover, HepG2 cells showed higher sensitivity to AST-3424. With increasing AST-3424 concentration, AKR1C3 protein expression level was downregulated significantly. The inhibition of AST-3424 was significantly higher than OXA, 5-Fu, Sor (sorafenib), and Apa (apatinib) in both HCC cells. AST-3424 monotherapy and combination therapy with OXA or 5-Fu all strongly inhibited the proliferation of HCC cells, blocked HCC cells in the S phase, promoted apoptosis induction, and suppressed the migration of HCC cells. Among them, the antitumor effect of AST-3424 in combination with OXA was obviously enhanced. Western blotting analysis demonstrated the regulation of P21, Bax, Caspase3, PARP, MMP-2, MMP-9, and p-Smad proteins in the presence of AST-3424 monotherapy and combination therapy with OXA or 5-Fu, indicating that its antitumor mechanisms may be associated with the regulation of the TGF-β signaling cascade.

**Conclusion:**

The *in-vitro* studies revealed that AST-3424 in combination with both OXA and 5-Fu showed an increased antitumor effect, and the combination with OXA resulted in a synergistic effect. Together with the *in-vitro* results, additional *in-vitro* and *in-vivo* studies are warranted to further certify its antitumor effects and explore more potential antitumor mechanisms.

## Introduction

The global incidence of primary liver cancer (PLC) is about 4.7% and the mortality rate is about 8.3%. It is the fifth most common and the third most lethal malignancy in the world. In China, there are about 410,000 new cases and 391,000 deaths every year, which together accounts for more than 50% of the global total (1). Most patients with liver cancer are in the middle and late stages when diagnosed and cannot be treated with surgery, and thus, systemic therapy is their main treatment. Systemic therapy usually includes systemic chemotherapy, targeted therapy, immunotherapy, and traditional Chinese medicine therapy. Notably enough, the last 5 years have witnessed an outstanding development of novel therapeutic options in hepatocellular carcinoma (HCC), including tyrosine kinase inhibitors (TKIs) (e.g., lenvatinib, cabozantinib, regorafenib) and anti-angiogenic monoclonal antibodies (ramucirumab) in first- and second-line settings, as well as immune checkpoint inhibitors (ICIs) and combinations of both strategies. Nonetheless, there are currently no validated predictive biomarkers able to guide treatment choice in this setting (2, 3). Traditionally, liver cancer is considered to be a tumor that is resistant to cytotoxic chemotherapeutic agents (4). With the development of systemically chemotherapeutic agents, especially in the area of research and application of new generation platinum agents, metronomic chemotherapy was introduced into oncology and proven in related studies; however, this concept is already obsolete and needs to be changed (5–7). As a result of the presence of basic liver diseases (including hepatitis, cirrhosis, liver dysfunction, and related complications), acquired drug-resistant side effects, and poor patient tolerance, the clinical application of systemic chemotherapy is still limited.

AKR1C3 is known to be overexpressed in a variety of malignant tumors, especially in liver cancer and prostate cancer (PC) (8–10). The high expression of AKR1C3 may lead not only to poor prognosis but also to resistance to a variety of antitumor therapies (including chemotherapy, radiotherapy, and targeted therapy) (11–14). Therefore, AKR1C3 as a new antitumor therapeutic target may be of great value. AST-3424, a chemically synthesized prodrug of a potent nitrogen mustard, is selectively cleaved to the bis-functional alkylating aziridine (OBI-2660) by AKR1C3, which is highly expressed in tumor cells resulting in a strong antitumor effect with less lymphocyte toxicity (15) and is a promising new agent for antitumor therapy. AST-3424 is currently being studied in a clinical trial (NCT03592264) for HCC and castration-resistant prostate cancer (CRPC).

In order to further verify the antitumor effects and mechanisms of AST-3424 in HCC, *in-vitro* studies were designed to explore its antitumor mechanism of action from multiple perspectives to provide a strong scientific basis for future studies.

## Materials and Methods

### Cell Culture

The HCC cell lines, HepG2 and PLC/PRF/5, were provided by the National Collection of Authenticated Cell Cultures (Shanghai, China). All cells were grown in DMEM containing 10% of fetal bovine serum (FBS) at 37°C in a 5% CO_2_ atmosphere.

### Agents and Antibodies

AST-3424 was provided by OBI Pharmaceuticals, Inc. (Asia). AST-3424 was dissolved in absolute ethyl alcohol at a concentration of 100 mM and stored at −20°C. Oxaliplatin (OXA) was purchased from Jiangsu Hengrui Medicine Co., Ltd. (Nanjing, China) and then dissolved in GS before use. 5-Fluorouracil (5-Fu) was purchased from Shanghai Xudong Medicine Co., Ltd. (Shanghai, China) and then dissolved in NS before use. The antibodies of Bax, β-catenin, Caspase3, Bcl-2, PARP, AKR1C3, P21, Smad2/3, MMP-2, and MMP-9 were purchased from GeneTex (Irvine, CA, USA). pSmad2/3 antibody was purchased from Cell Signaling Technology (Beverly, MA, USA). Tubulin antibody and TGF-β growth factor were purchased from Jiangsu Keygen Biotech Corp., Ltd. (Nanjing, China).

### Cell Proliferation Analysis

Cell growth and viability were detected using the Cell Counting Kit-8 (CCK-8) assay. HepG2 and PLC/PRF/5 cells were cultured overnight at 2 × 10^3^ cells/well in 96-well plates, respectively. Subsequently, the original medium was replaced by a complete medium containing AST-3424, OXA, 5-Fu, or their combination in different concentrations. After 48 h of incubation, CCK-8 was added to each well. The plates were further incubated at 37°C for 2 h and the optical density (OD) (260 nm) of the samples was measured with an enzyme-linked instrument (Bio-Rad, Hercules, California, USA) at 450 nm. Cell viability was calculated by the following equation: viability = (the OD of the treatment group/the OD of the control group) × 100%.

### Drug Combination Therapy Effect Evaluation

A coefficient of drug interaction (CDI) value was used to investigate the drug interaction between AST-3424 and OXA or 5-Fu. The CDI value was calculated by the following equation: CDI = EAB/(EA × EB). EAB is the absorbance ratio (260 nm) of the two-drug combination group and the control group and EA or EB is the absorbance ratio of the single-drug group and the control group.

### Colony Formation Assay

HepG2 cells were incubated at 500 cells/well in six-well plates and then underwent different treatments for 48 h. Subsequently, the previous medium was replaced by a complete medium for further 10 days of incubation. The colonies were stained with 1% crystal violet for 20 min and then washed with PBS before use. After drying, the visible colonies were photographed and counted.

### Apoptosis Assay

Following treatment with AST-3424, OXA, or 5-Fu monotherapy or combination therapy for 48 h, the HepG2 cells were trypsinized without ethylene diamine tetraacetic acid (EDTA). After washing with cold phosphate-buffered saline (PBS) twice, followed by centrifugation at 3,000 rpm for 2 min, at least 1 × 10^5^ cells/ml were collected and resuspended in buffer. An Annexin V-FITC/PI-staining kit (UE Biotechnology Co., Watertown, USA) was used to stain the cells. The percentage of apoptotic cells was quantified using a flow cytometer (Becton Dickinson and Co., Franklin Lakes, NJ, USA).

### Cell Cycle Analysis

Following treatment with AST-3424, OXA, or 5-Fu monotherapy or combination therapy for 48 h, the HepG2 cells were trypsinized without EDTA and washed twice with cold PBS followed by centrifugation at 3,000 rpm for 2 min. The 1 × 10^6^ cells/ml were resuspended in 80% of precooled alcohol to make single-cell suspensions and stored at 4°C overnight. A cell cycle staining kit (KenGen Biotechnology Co., Nanjing, China) was used to stain the cells. After being stained for 30 min, the samples were examined by a flow cytometer, and the results were analyzed by the FlowJo software (Becton, Dickinson and Co., Ashland, OR, USA).

### Cell Migration and Invasion Assay

Cell migration and invasion assays were performed by using Transwell chambers with 8.0-μm pore size membranes. Cells were treated with AST-3424, OXA, or 5-Fu monotherapy or combination therapy for 48 h and collected in advance. Subsequently, cells were resuspended in a medium without FBS and 200 μl (5 × 10^5^ cells/ml) was added to the upper chamber, while 600 μl of the medium with 10% FBS was added to the lower chamber. After being incubated for 12 h, cells on the upper surface were wiped away, and those on the lower surface were stained with 0.1% crystal violet. Lastly, photomicrographs were taken, and the number of migrated cells was calculated in three random microscope fields. The cell invasion assay was performed as described above for the cell migration assay, except for the upper surface of the Transwell chamber that was coated with diluted Matrigel.

### Western Blotting

Procedures for Western blotting have been described previously (16). Cells were treated with AST-3424, OXA, or 5-Fu monotherapy or combination therapy for 48 h. The protein samples were premixed with loading buffer and then separated and transferred to polyvinylidene fluoride (PVDF) membranes in 10% SDS-PAGE and 5% BSA used as blocking buffer. Subsequently, the membranes were incubated with specific primary antibodies and the bands were visualized using enhanced chemiluminescence.

### Statistical Analysis

SPSS 21.0 and GraphPad Prism 8 software were used for statistical analysis and data presentation. Comparisons among the different groups were conducted using Student’s *t*-tests. Data were expressed as the mean ± standard deviation of three repeated experiments. A level of *p <*0.05 was accepted as statistically significant.

## Results

### Inhibition of AST-3424, Chemotherapeutics, and Targeted Drugs in Different HCC Cells

**Figure 1:**
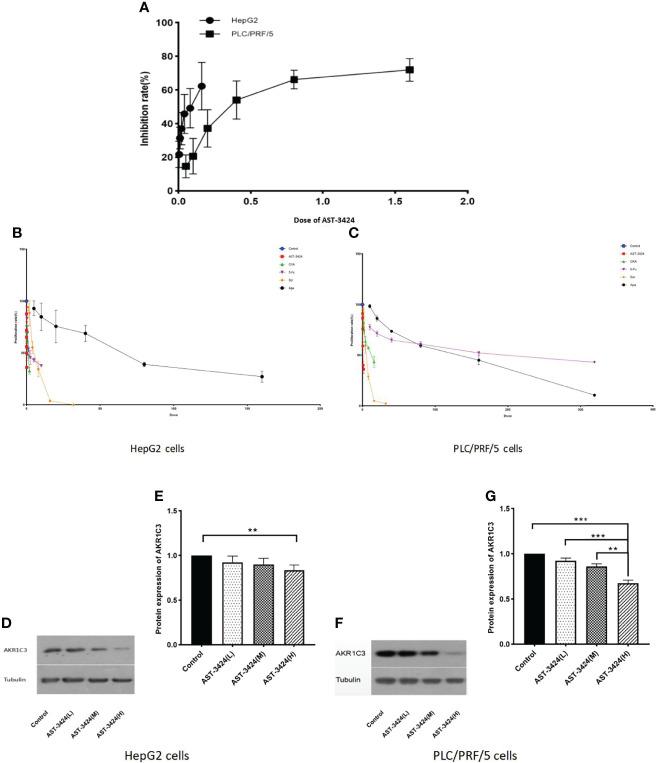
Inhibition of AST-3424 in HepG2 and PLC/PRF/5 cells. **(A)** Inhibition at different concentrations of AST-3424 (0.005–0.16 μM) in HepG2 and AST-3424 (0.05–1.6 μM) in PLC/PRF/5 cells. **(B)** The effect of different concentrations of AST-3424 (0.005–0.16 μM), oxaliplatin (OXA) (0.0625–2 μg/ml), 5-fluorouracil (5-Fu) (0.3125–10 μg/ml), sorafenib (Sor) (1–32 μg/ml), and apatinib (Apa) (5–160 μg/ml) treated for 48 h in HepG2 cells. **(C)** The effect of different concentrations of AST-3424 (0.05–1.6 μM), OXA (0.5–16 μg/ml), 5-Fu (10–320 μg/ml), Sor (1–32 μg/ml), and Apa (10–320 μg/ml) treated for 48 h in PLC/PRF/5 cells. **(D–G)** AKR1C3 protein expression levels in HepG2 and PLC/PRF/5 cells following treatment with different concentrations of AST-3424. All data are displayed as the mean ± SEM of biological triplicates; *p < 0.05, **p < 0.01, ***p < 0.001.

We evaluated the inhibition of AST-3424 in different HCC cells by the CCK-8 assay. Based on a previous study (8), we selected the HepG2 and PLC/PRF/5 cells for explicitly expressing the AKR1C3 protein. The results showed that the inhibiting effects of AST-3424 were significant in both HCC cells. Moreover, HepG2 cells showed higher sensitivity to AST-3424, while PLC/PRF/5 cells showed relative drug resistance (**Figure 1A**). Meanwhile, we selected two chemotherapeutics (OXA, 5-Fu) and two targeted drugs [sorafenib (Sor), apatinib (Apa)] to evaluate the inhibition between them in different HCC cells relatively (**Figures 1B, C**). We found that the proliferation inhibition rate of AST-3424 was significantly higher than that of the other drugs in both HCC cells. In both cells, AKR1C3 protein expression was significantly downregulated with increasing AST-3424 concentrations (*p *< 0.01, **Figures 1D–G**) and showed a significant negative correlation (**Figures 1D–G**). These results suggest that AKR1C3 expression may be a potential influencing factor in the antitumor effect of AST-3424. In this study, we aimed to evaluate the *in-vitro* inhibiting effects of AST-3424 monotherapy and combination therapy with OXA or 5-Fu in HCC cells.

### The *In-Vitro* Inhibiting Effects of AST-3424 Monotherapy and Combination Therapy With OXA or 5-Fu in HCC Cells

**Figure 2:**
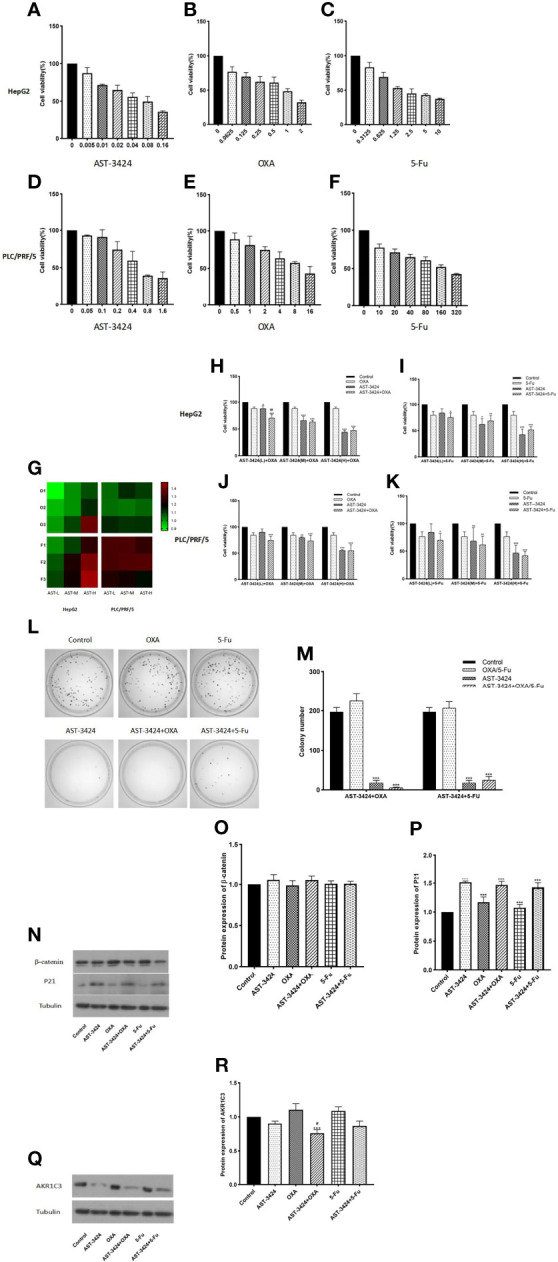
The effect of treatment with AST-3424, OXA, or 5-Fu alone or in combination on cell proliferation and colony formation in HepG2 and PLC/PRF/5 cells. **(A–C)** Treatment with AST-3424 (0.005–0.16 μM), OXA (0.0625–2 μg/ml), or 5-Fu (0.3125–10 μg/ml) alone for 48 h in HepG2 cells. **(D–F)** Treatment with AST-3424 (0.05–1.6 μM), OXA (0.5°16 μg/ml), or 5-Fu alone (10–320 μg/mL) for 48 h in PLC/PRF/5 cells. **(G)** Heat graph of coefficient of drug interaction (CDI) values in HepG2 and PLC/PRF/5 cells [AST-3424 (T1-H 0.005 μM; T2-H 0.01 μM; T3-H 0.05 μM) combined with OXA (O1 0.02 μg/ml; O2 0.15 μg/ml; O3 1 μg/ml) or 5-Fu (F1 0.3125 μg/ml; F2 0.625 μg/ml; F3 1.25 μg/ml) for 48 h in HepG2 cells and AST-3424 (T1-P 0.1 μM; T2-P 0.3 μM; T3-P 0.6 μM) combined with OXA (O1 0.5 μg/ml; O2 3 μg/ml; O3 9 μg/ml) or 5-Fu (F1 2 μg/ml; F2 20 μg/ml; F3 160 μg/ml) for 48 h in PLC/PRF/5 cells)]. A CDI >1 indicates antagonism; CDI <1 means synergy; CDI = 1 indicates additive effects. **(H, I)** Cell proliferation ability was assessed after HepG2 cells were treated with AST-3424-L (0.005 μM), AST-3424-M (0.01 μM), AST-3424-H (0.05 μM), OXA (0. 02 μg/ml), and 5-Fu (0.3125 μg/mL) alone or in combination for 48 h. **(J, K)** Cell proliferation ability was assessed after PLC/PRF/5 cells were treated with AST-3424-L (0.1 μM), AST-3424-M (0.3 μM), AST-3424-H (0.6 μM), OXA (0.5 μg/ml), and 5-Fu (2 μg/ml) alone or in combination for 48 h. **(L, M)** The inhibiting effects on colony formation following monotherapy or combination therapy were shown in HepG2 cells, and the colony numbers were calculated. **(N–P)** The protein expressions of β-catenin and P21 in HepG2 cells after 48 h of treatment with AST-3424, OXA, and 5-Fu alone or in combination were measured using Western blotting, and the relative optical density of the protein expression was analyzed using ImageJ. **(Q, R)** AKR1C3 protein expression levels in HepG2 and PLC/PRF/5 cells following treatment with AST-3424, OXA, or 5-Fu alone or in combination. All data are expressed as the mean ± SEM of three separate experiments. *p < 0.05, **p < 0.01, ***p < 0.001, compared to the control group; ^#^ *p *< 0.05, ^##^ *p *< 0.01, ^###^ *p *< 0.001, the combination group compared to the AST-3424 monotherapy group.

In order to evaluate the *in-vitro* effects of AST-3424 monotherapy and combination therapy with OXA or 5-Fu on the proliferation activity of HepG2 and PLC/PRF/5 cells, we used the CCK-8 assay. The results showed that the proliferation inhibition rate of AST-3424 (0.005–0.16 μM) in HepG2 cells was 12.68%–63.99% (**Figure 2A**). The proliferation inhibition rate of AST-3424 (0.05–1.6 μM) in PLC/PRF/5 cells was 6.81%–64.13% (**Figure 2D**). These results suggested that AST-3424 exhibited strong cytotoxicity and concentration-dependent inhibition of proliferation in both HepG2 and PLC/PRF/5 cells. Especially in HepG2 cells, IC_50_ values were in the lower nanomolar range. Based on these data, AST-3424 concentrations corresponding to proliferation inhibition rates of 10%–15%, 30%, and 50% were selected for the combination study, and the synergistic antiproliferation effect of the combinations was investigated by CDI values. Concentrations with 10%–15% inhibition rate of OXA (0.02 μg/ml) and 5-Fu (0.3125 μg/ml) in combination with AST-3424 may have synergistic effects (**Figures 2B, C, G**). Compared to the control group, different concentrations of AST-3424 in combination with OXA (0.02 μg/ml) or 5-Fu (0.3125 μg/ml) significantly inhibited tumor cell proliferation (*p *< 0.05; **Figures 2H–K**). With increasing concentrations of AST-3424, the inhibition effect of proliferation in combination with OXA or 5-Fu was slowly approaching that of AST-3424 monotherapy or might even be weaker than AST-3424 monotherapy (**Figures 2H–K**). Based on the CDI values (**Tables 1**, **2**), AST-3424 in combination with OXA showed synergistic antiproliferative effect. As the AST-3424 concentrations increased, the synergistic antiproliferative effect was reduced, while AST-3424 in combination with 5-Fu showed antagonism. Based on these data, we selected HepG2 cells that were more sensitive to AST-3424 as well as the drug concentrations of AST-3424 (0.05 μM), OXA (0.02 μg/ml), and 5-Fu (0.3125 μg/ml) for the subsequent mechanism-related exploration experiments. In addition, these results were verified by clone formation experiments. Compared to the control group, AST-3424 in combination with OXA or 5-Fu significantly inhibited cell clone formation (*p *< 0.01, **Figures 2L,M**), but the proliferation inhibition of the combination of AST-3424 with OXA was more significant. Compared to AST-3424 monotherapy, the inhibition of cell clone formation was enhanced by the combination with OXA, whereas the inhibition of cell clone formation was weakened by the combination with 5-Fu (**Figures 2L, M**). The results of the clone formation expriments were basically consistent with the cell proliferation studies.

**Table 1:** Fraction affected level (Fa) of AST-3424, OXA, and 5-Fu alone or in combination and CDI values of the combination in HepG2 cells.

AST-3424 (μM)	OXA (μg/ml)	5-Fu (μg/ml)	Fa of AST-3424	Fa of OXA	Fa of AST + OXA	CDI	Fa of 5-Fu	Fa of AST + Fu	CDI
0.005	0.02	0.3125	11.95 ± 5.10	11.57 ± 2.69	34.66 ± 0.139	0.893	20.26 ± 6.71	24.72 ± 8.89	1.197
0.01			33.99 ± 7.33		43.84 ± 0.103	0.941		31.35 ± 10.10	1.567
0.05			55.85 ± 2.96		53.95 ± 0.115	1.006		48.50 ± 6.26	2.144

**Table 2:** Fraction affected level (Fa) of AST-3424, OXA, and 5-Fu alone or in combination and the CDI values of the combination in PLC/PRF/5 cells.

AST-3424 (μM)	OXA (μg/ml)	5-Fu (μg/ml)	Fa of AST-3424	Fa of OXA	Fa of AST + OXA	CDI	Fa of 5-Fu	Fa of AST + Fu	CDI
0.1	0.5	2	10.25 ± 5.12	16.11 ± 3.67	33.17 ± 5.72	0.975	18.81 ± 3.47	26.53 ± 6.34	1.112
0.3			21.48 ± 4.16		39.41 ± 12.46	1.089		33.65 ± 7.54	1.199
0.6			46.44 ± 2.64		57.46 ± 10.41	1.168		57.23 ± 6.32	1.323

In order to explore the potential mechanism of AST-3424 monotherapy and combination therapy with OXA or 5-Fu to inhibit proliferation, we detected the expression of β-catenin and P21 proteins that were closely related to cell proliferation by a Western blot assay. At the same time, the expression of AKR1C3 protein was also detected. Compared to the control group, AST-3424 monotherapy and combination therapy with OXA or 5-Fu significantly upregulated P21 protein expression (*p *< 0.01, **Figures 2N, P**), and there was no significant difference in β-catenin protein expression (*p* > 0.05, **Figures 2N, O**). Compared to AST-3424 monotherapy, the protein expression of P21 and β-catenin was not significantly different from combination therapy (*p* > 0.05, **Figures 2N, P**). However, compared to the control group, AST-3424 monotherapy and combination therapy with OXA or 5-Fu downregulated the expression of the AKR1C3 protein. Among them, AST-3424 in combination with OXA significantly downregulated AKR1C3 protein expression (*p *< 0.01, **Figures 2Q, R**). These results suggested that AST-3424 monotherapy and combination therapy with OXA or 5-Fu could inhibit cell proliferation. Moreover, the inhibitory effect of the combination with OXA was significantly enhanced, suggesting a synergistic effect. The mechanism may be related to the upregulation of P21 protein and may not be associated with β-catenin protein expression.

### AST-3424 Monotherapy and Combination Therapy With OXA or 5-Fu Can Promote Cell Apoptosis Induction and Cell Cycle Arrest

**Figure 3:**
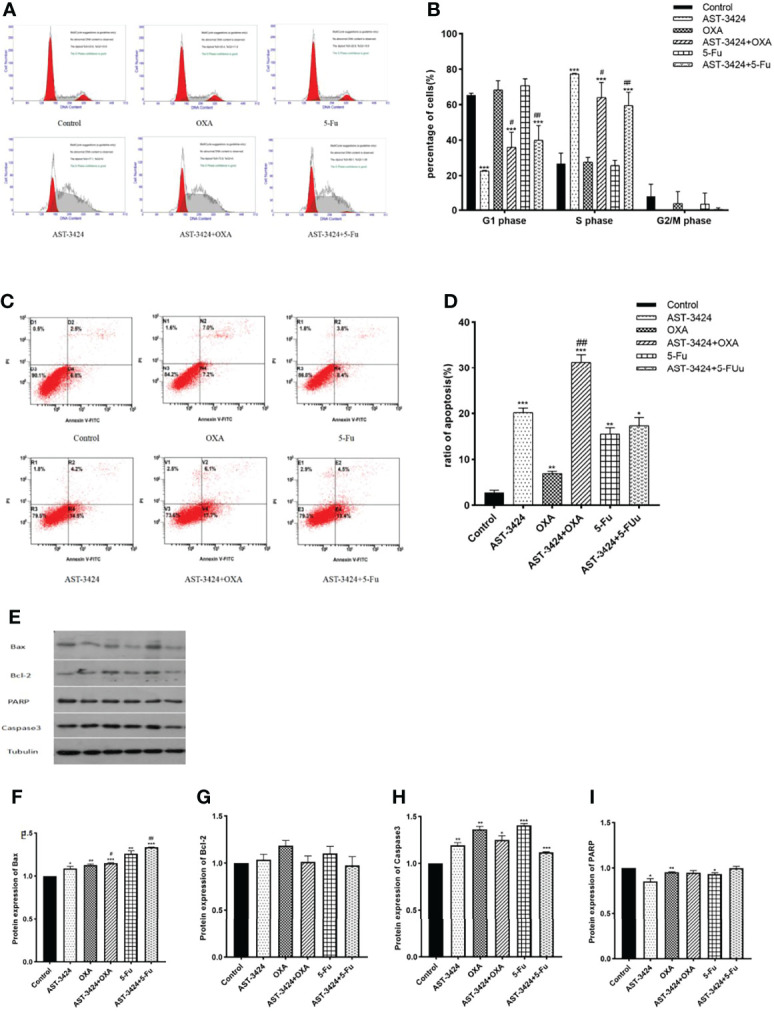
Cell cycle arrest effect and apoptosis induction effect of AST-3424, OXA, and 5-Fu as a single agent or in combination with AST-3424 on HepG2 cells. **(A)** DNA content-based cell cycle of HepG2 cells after treatment with AST-3424 (0.05 μM), OXA (0.02 μg/ml), and 5-Fu (0.3125 μg/ml) alone or in combination was analyzed using flow cytometry. **(B)** Percentage of cells in the S phase of HepG2 cells after treatment with AST-3424 (0.05 μM), OXA (0.02 μg/ml), and 5-Fu (0.3125 μg/ml) alone or in combination with AST-3424. **(C, D)** Flow cytometry histograms of HepG2 cells after 48 h of treatment with AST-3424 (0.05 μM), OXA (0.02 μg/ml), and 5-Fu (0.3125 μg/ml) alone or in combination with AST-3424; the samples were detected using AV/PI double-staining method. The percentage of apoptotic cells was calculated, and the results are shown in the bar graph. **(E–I)** The expression levels of anti-apoptotic proteins, Bax, Bcl-2, Caspase3, and PARP, in HepG2 cells of different treatments were detected by Western blot, and the relative optical densities of the proteins were analyzed by ImageJ. All results are expressed as mean ± SEM of three independent experiments. *p < 0.05, **p < 0.01, ***p < 0.001, compared to the control group; ^#^ *p *< 0.05, ^###^ *p *< 0.01, ^###^ *p *< 0.001, the combination group compared to the AST-3424 monotherapy group.

To determine whether AST-3424 monotherapy and combination therapy with OXA or 5-Fu affected the cell cycle of HepG2 cells, flow cytometry was used to detect the percentage of cells in different phases. Compared to the control group, the proportion of cells in the S phase increased significantly with AST-3424 monotherapy (26.80% to 77.39%) and combination therapy with OXA (27.65% to 64.03%) or 5-Fu (25.70% to 59.57%) (*p *< 0.01, **Figures 3A, B**). Compared to AST-3424 monotherapy, the proportion of cells in the G1 phase increased significantly after treatment with AST-3424 in combination with OXA or 5-Fu therapy (*p *< 0. 05, **Figures 3A, B**). These results suggested that AST-3424 monotherapy and combination therapy with OXA or 5-Fu could block the cells in the S phase.

The inducing of apoptosis after treatment with AST-3424 monotherapy and combination therapy with OXA or 5-Fu was detected by flow cytometry. The results showed that compared to the control group, AST-3424 monotherapy and combination therapy with OXA or 5-Fu could significantly induce apoptosis of HepG2 cells (*p *< 0.05, **Figures 3C, D**). Compared to AST-3424 monotherapy, AST-3424 in combination with OXA significantly enhanced the apoptosis-inducing effect (*p *< 0.01), and although the apoptosis-inducing effect of AST-3424 in combination with 5-Fu was enhanced, it was not significantly different (*p* > 0.05, **Figure 3D**). These results suggested that AST-3424 monotherapy and combination therapy with OXA or 5-Fu could promote apoptosis induction, and the combination with OXA had a synergistic effect. In order to further understand the mechanism of apoptosis induced by the treatment with AST-3424 monotherapy and combination therapy with OXA or 5-Fu, we detected the expression of four apoptotic proteins, Bax, Bcl-2, Caspase3, and PARP. Compared to the control group, the results showed that AST-3424 monotherapy and combination therapy with OXA or 5-Fu significantly upregulated the expression of Bax and Caspase3 proteins (*p *< 0.05) and slightly upregulated the expression of Bcl-2 protein (*p* > 0.05). AST-3424 monotherapy significantly downregulated the expression of the PARP protein (*p *< 0.05), but it was not affected in the combination groups (*p* > 0.05, **Figures 3E–I**). Compared to AST-3424 monotherapy, Bax protein expression was upregulated after combination therapy, but it was not significant (*p* > 0.05, **Figure 3F**). Caspase3 protein expression was slightly upregulated in combination with OXA and slightly downregulated in combination with 5-Fu (*p* > 0.05, **Figure 3H**). PARP protein expression was upregulated, but the difference was not statistically significant (*p* > 0.05, **Figure 3I**). These results suggested that AST-3424 monotherapy and combination therapy with OXA or 5-Fu may induce apoptosis by upregulating Bax and Caspase3 proteins and downregulating the PARP protein.

### AST-3424 Monotherapy and Combination Therapy With OXA or 5-Fu Inhibited HepG2 Cell Metastasis

**Figure 4:**
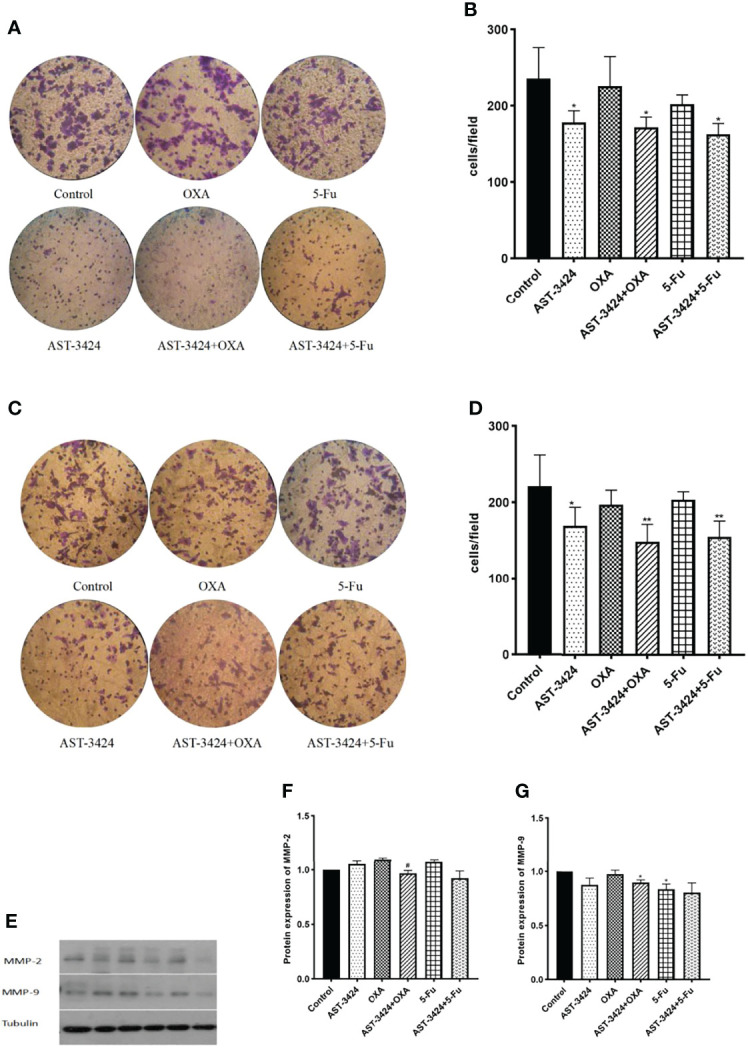
Effects of treatment with AST-3424, OXA, and 5-Fu alone or in combination with AST-3424 migratory and invasive abilities of HepG2 cells. **(A, B)** Representative images of Transwell assay for invasion using Transwell chambers with Matrigel coating; HepG2 cells that had penetrated through the filter after treatment with AST-3424, OXA, and 5-Fu monotherapy or in combination for 48 h were calculated. **(C, D)** Representative images of Transwell assay for migration using Transwell chambers without Matrigel coating; HepG2 cells that had migrated through the filter after treatment with AST-3424, OXA, and 5-Fu monotherapy or in combination for 48 h were calculated. **(E–G)** Western blot results of MMP-2 and MMP-9 protein expression after treatment with AST-3424, OXA, and 5-Fu alone or in combination, and relative optical density of the proteins was analyzed based on ImageJ. All results are expressed as mean ± SEM of three independent experiments. *p < 0.05, **p < 0.01, ***p < 0.001, compared to the control group; ^#^ *p *< 0.05, ^##^ *p *< 0.01, ^###^ *p *< 0.001, the combination group compared to the AST-3424 monotherapy group.

To investigate whether AST-3424 monotherapy and combination therapy with OXA or 5-Fu could inhibit the metastasis of HepG2 cells, we performed Transwell cell migration and invasion assay. The results showed that, compared to the control group, AST-3424 monotherapy and combination therapy with OXA or 5-Fu significantly inhibited the migration and invasion of HepG2 cells (*p *< 0.05), and there was no significant difference between the AST-3424 monotherapy and the combination therapy (*p* > 0.05, **Figures 4A–D**). Subsequently, we detected MMP-2 and MMP-9 protein expression associated with tumor metastatic ability to explore possible mechanisms. The results showed that, compared to the control group, AST-3424 monotherapy and combination therapy with OXA or 5-Fu could downregulate MMP-9 protein expression, but only the result of AST-3424 in combination with OXA had a significant difference (*p *< 0.05, **Figures 4E–G**); AST-3424 monotherapy could slightly upregulate the expression of the MMP-2 protein and the combination therapy could slightly downregulate the expression of the MMP-2 protein (*p* > 0.05, **Figures 4E–G**). Compared to AST-3424 monotherapy, the effect of downregulation of MMP-2 protein expression in combination with the AST-3424 group was enhanced, but the difference was not statistically significant (*p* > 0.05, **Figures 4E–G**). Although the effect of downregulation of MMP-9 protein expression in combination with the 5-Fu group was slightly enhanced, the difference was not statistically significant (*p* > 0.05, **Figures 4E–G**). However, in combination with the OXA group, the expression of the MMP-9 protein was slightly upregulated (*p* > 0.05, **Figures 4E–G**). These results suggested that AST-3424 monotherapy and combination therapy with OXA or 5-Fu could inhibit the migration and invasion of tumor cells, and the mechanism may be related to the downregulation of MMP-9 proteins.

### Inhibition of TGF-β Activation by AST-3424 Monotherapy and Combination Therapy With OXA or 5-Fu

**Figure 5:**
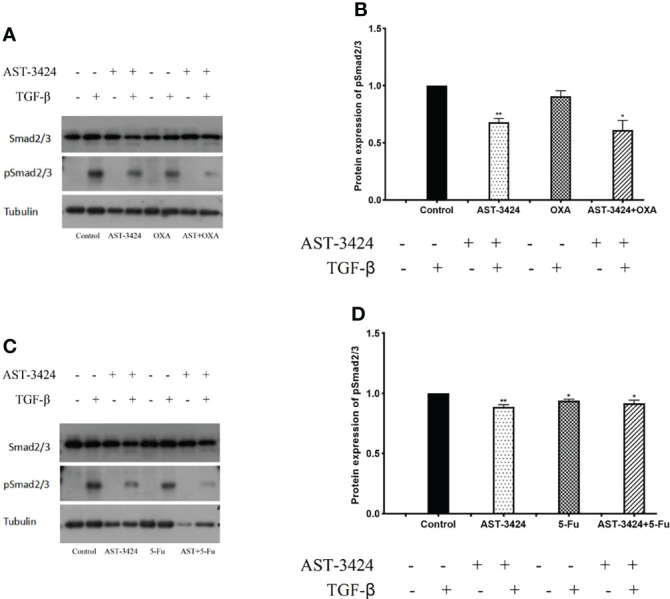
AST-3424 synergized with OXA promoting inhibition in HepG2 cells. p-Smad2/3 protein expression was detected using Western blot after 48 h of treatment with AST-3424 and OXA or 5-Fu alone or in combination with AST-3424 and 4 h of exposure to TGF-β **(A–D)**. The relative optical density of the proteins was analyzed by ImageJ. All results are expressed as mean ± SEM of three independent experiments. *p < 0.05, **p < 0.01, ***p < 0.001, compared to the control group; ^#^ *p *< 0.05, ^###^ *p *< 0.01, ^###^ *p *< 0.001, the combination group compared to the AST-3424 monotherapy group.

Since TGF-β signaling pathways are continuously activated in HCC and closely related to their proliferation, apoptosis, and metastasis, we investigated the effects of AST-3424 monotherapy and combination therapy with OXA or 5-Fu on the TGF-β signaling pathway by using its activated form, p-Smad2/3 (tyrosine phosphorylated Smad 2/3). After being prestarved for 24 h, HepG2 cells were treated with AST-3424 monotherapy and combination therapy with OXA or 5-Fu for 48 h and then continued to be stimulated to Smad2/3 phosphorylation for 4 h by TGF-β growth factor. The expression of total Smad2/3 and p-Smad2/3 proteins was detected by Western blot. The results showed that, compared to the control group, AST-3424 monotherapy and combination therapy with OXA and 5-Fu significantly decreased the expression of the p-Smad2/3 protein (*p *< 0.05), while the total Smad2/3 protein was not affected (**Figures 5A–D**). Compared to AST-3424 monotherapy, the downregulation of the p-Smad2/3 protein was more pronounced in AST-3424 in combination with OXA, but it was not significant (*p* > 0.05, **Figures 5A, B**). The p-Smad2/3 protein was slightly upregulated in the group of AST-3424 in combination with 5-Fu, but it was not significant (*p* > 0.05, **Figures 5C, D**). These results suggested that AST-3424 monotherapy and combination therapy with OXA or 5-Fu could reduce the effect of proliferation by inhibiting the TGF-β signaling pathway, and the inhibition effect was significant only in the combination of AST-3424 and OXA.

## Discussion

In recent years, along with advances in molecular biology and molecular immunology research, more and more targeted drugs and immune checkpoint inhibitors have been approved for the clinical treatment of liver cancer, but their efficacy is still limited. Pretreatment screening of patients and acquired drug resistance and adverse reactions after treatment are still the challenges and urgent problems to be solved with currently available treatments. In early years, it was found that due to the high heterogeneity and complexity of liver cancer, there was a strong resistance to traditional cytotoxic chemotherapeutic drugs (such as 5-Fu and doxorubicin). However, recent studies have shown that systemic chemotherapy with OXA in the treatment of advanced HCC can improve objective efficacy and benefit survival (17–19). Meanwhile, systemic chemotherapy with OXA in combination with other treatments can synergize the effect (20–22). Therefore, systemic chemotherapy has become an important treatment for advanced liver cancer.

Cytotoxic drugs represented by alkylating agents are classical traditional chemotherapeutic agents first used in the systemic therapy of tumors. However, due to the lack of selectivity in their anticancer effect, they can cause damage to normal tissues and organs as well as functional damage while killing tumor cells. A potential innovative therapy is to use enzyme activity to activate novel prodrugs to alkylating agents, which not only can be activated and retained in target cancer cells but also exhibit good safety, selectivity, and efficacy. Although PR-104 and CTX (cyclophosphamide) are both prodrugs that are activated *in vivo*, their efficacy and targeting are limited, and the toxicity is obvious (23–25). In contrast, AST-3424, as a prodrug of an inactivated nitrogen mustard, is selectively an effective DNA alkylated compound, OBI-2660, in cells, which is present as a polar salt at pH 7.4 and unable to penetrate the plasma membrane. This property may lead to the reduction of systemic and/or bystander toxicity (15).

Abnormal cell proliferation caused by many factors will promote the occurrence and development of tumors. Changing the cell cycle mechanism of tumors is an important means to maintain its unlimited proliferation ability and also an important cause of tumor growth and increase of tumor load *in vivo*. In addition, clinical deaths that are associated with liver cancer are also caused by metastatic lesions (26). Therefore, the occurrence and development of tumors are often inhibited to prolong the survival time of patients with advanced liver cancer by inhibiting cell proliferation, inducing cell apoptosis, affecting the tumor cell cycle, and weakening the invasion and metastasis ability of liver cancer (27). In this study, we found that AST-3424 monotherapy and combination therapy with OXA or 5-Fu exhibited strong cytotoxicity *in vitro* and significant cell cycle arrest in HCC cell lines. The role of AST-3424 in combination with OXA showed a more significant synergistic effect. These results suggested that AST-3424, a cycle-specific antitumor drug having a potential mechanism of action on DNA, can kill a large number of cells in the proliferative stage. In combination with OXA (a periodic specific antitumor drug), it can further kill the remaining tumor cells, playing a synergistic antitumor effect. On the one hand, it is considered that the cells can be blocked in the S phase and promote apoptosis induction by upregulating the expression of P21 and Bax proteins or directly promote apoptosis induction by upregulating the expression of the Caspase3 protein to degrade the PARP protein. On the other hand, the downregulation of the MMP-9 protein may weaken the degradation of the IV-type collagen, which is the main component of the extracellular matrix and basement membrane. It can maintain the integrity of the basement membrane by inhibiting the metastasis of tumor cells.

It is known that the TGF-β signaling pathway can promote tumor angiogenesis, induce invasion and metastasis by inducing the proliferation and survival of HCC cells and promoting epithelial–mesenchymal transformation (EMT), and create a favorable immune microenvironment for tumor growth to continuously promote tumor development (28). Moreover, the TGF-β signaling pathway can also participate in the activation of various invasive and metastatic pathways through Smad2/3-dependent and Smad2/3-non-dependent signaling mechanisms (29). In this study, we found that AST-3424 monotherapy and combination therapy with OXA or 5-Fu can inhibit the activation of the TGF-β signaling pathway to block the development of tumors. These results suggest that TGF-β is an important signaling pathway to promote the occurrence and development of liver cancer. Meanwhile, the strong *in-vitro* cytotoxicity of AST-3424 may be related to blocking the activation of this pathway. On the one hand, it may upregulate the expression of P21 by inhibiting the activation of Smad2/3 (pSmad2/3) to make tumor cells stagnate in the S phase and, thus, inhibit their proliferation. On the other hand, MMP-9/MMP-2 protein expression may be downregulated by inhibiting the activation of Smad2/3 (pSmad2/3), thereby inhibiting the occurrence of EMT and weakening the metastatic ability of tumor cells.

Of course, our study still has some limitations. We need to increase the *in-vivo* experiments to make the experimental results more reliable and increase the efficacy comparison of different AKR1C3 expression cell lines to expand the research direction, which can be further improved in subsequent experiments.

## Summary

In summary, AST-3424 monotherapy and combination therapy with OXA or 5-Fu can exert an *in-vitro* antitumor effect on liver cancer cells by inhibiting cell proliferation, inducing cell stagnation in the S phase, promoting cell apoptosis induction, inhibiting cell metastasis, etc. Among them, AST-3424 in combination with OXA exhibited a strong synergistic effect. The results of this study provide an important experimental basis for clearing the antitumor effect of AST-3424 and for further understanding its possible molecular mechanism, which is worthy of further study.

## Data Availability Statement

The original contributions presented in the study are included in the article/**Supplementary Material**. Further inquiries can be directed to the corresponding author.

## Author Contributions

YZ and SQ designed and conducted the experiments, analyzed the data for statistical significance, and wrote the paper. JC and YS designed and performed the experiments. YL and JD revised the manuscript. All authors contributed to the article and approved the submitted version.

## Conflict of Interest

Author JD was employed by Ascentawits Pharmaceuticals, Ltd.

The remaining authors declare that the research was conducted in the absence of any commercial or financial relationships that could be construed as a potential conflict of interest.

## Publisher’s Note

All claims expressed in this article are solely those of the authors and do not necessarily represent those of their affiliated organizations, or those of the publisher, the editors and the reviewers. Any product that may be evaluated in this article, or claim that may be made by its manufacturer, is not guaranteed or endorsed by the publisher.

## Abbreviations

PLC, primary liver cancer; HCC, hepatocellular carcinoma; OXA, oxaliplatin; 5-Fu, 5-fluorouracil; Apa, apatinib; Sor, sorafenib; AKR1C3, 17beta-hydroxysteroid dehydrogenase type 5; PC, prostate cancer; CRPC, castration-resistant prostate cancer; FBS, fetal bovine serum; CCK-8, Cell Counting Kit-8; OD, optical density; CDI, coefficient of drug interaction; PBS, phosphate-buffered saline; EDTA, ethylene diamine tetraacetic acid; PVDF, polyvinylidene fluoride; TGF-β, transforming growth factor-beta; Dox, doxorubicin.
