# Supplementary material for: The In-Vitro Antitumor Effects of AST-3424 Monotherapy and Combination Therapy With Oxaliplatin or 5-Fluorouracil in Primary Liver Cancer
Source: Front Oncol. 2022 Jul 22;12:885139. doi: 10.3389/fonc.2022.885139 (PMC9354847; doi:10.3389/fonc.2022.885139)
Supplement: Supplementary file 1 [file DataSheet_1.pdf]

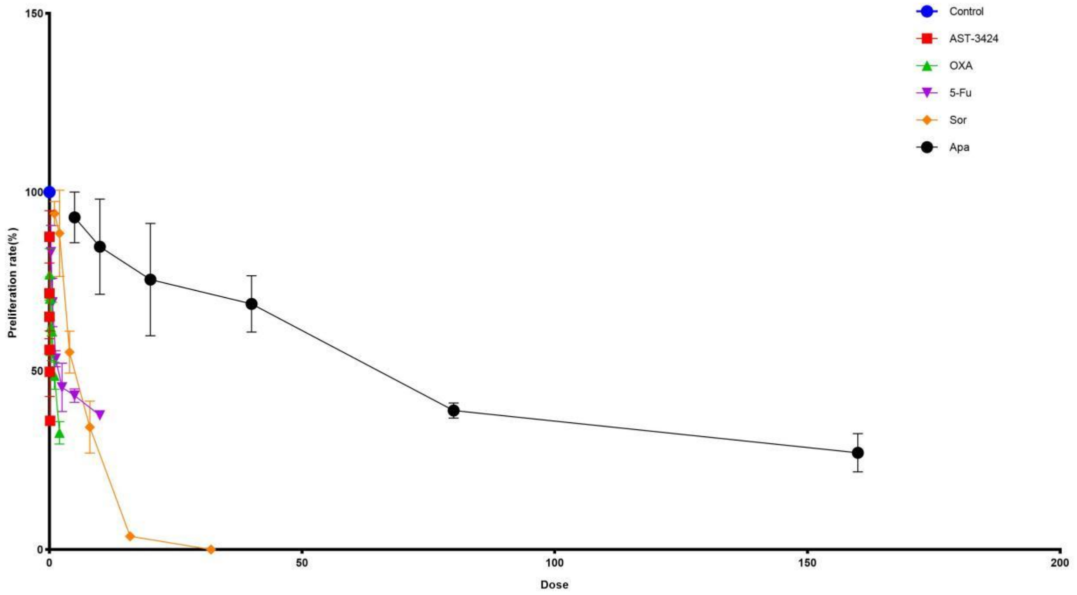

FIGURE | The effect at different concentrations of AST-3424(0.005-0.16 μM), OXA (0.0625-2μg/mL), 5-Fu(0.3125-10 μg/mL), Sor(1-32 μg/mL) and Apa(5-160 μg/mL) treated for 48 h in HepG2 cells. All data are expressed as the mean ± SEM of three separate experiments.

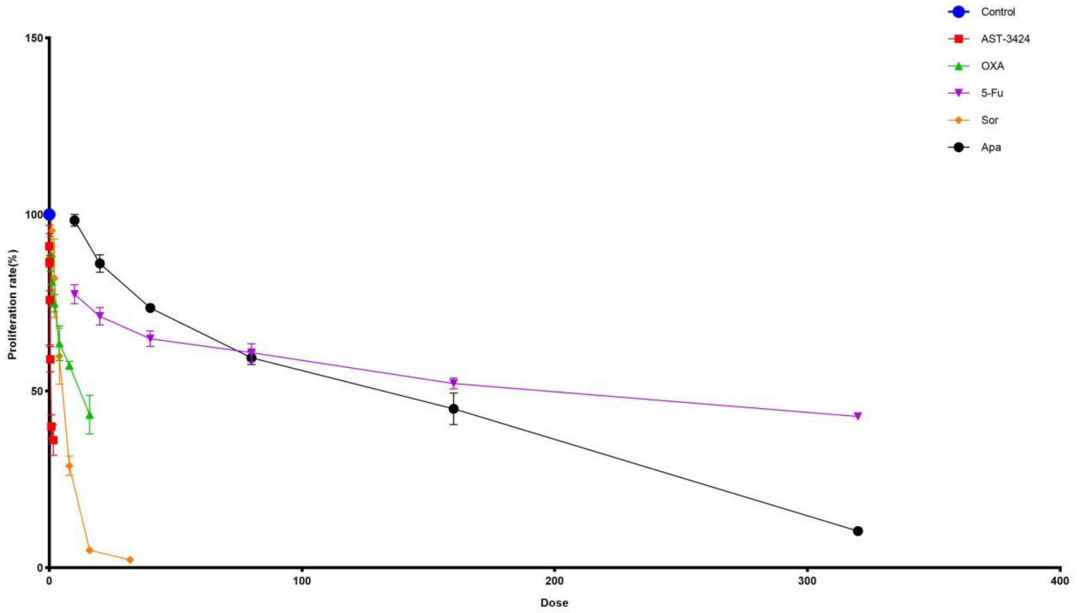

FIGURE | The effect at different concentrations of AST-3424(0.05-1.6 μM), OXA (0.5-16μg/mL), 5-Fu(10-320 μg/mL), Sor(1-32 μg/mL) and Apa(10-320 μg/mL) treated for 48 h in PLC/PRF/5 cells. All data are expressed as the mean ± SEM of three separate experiments.

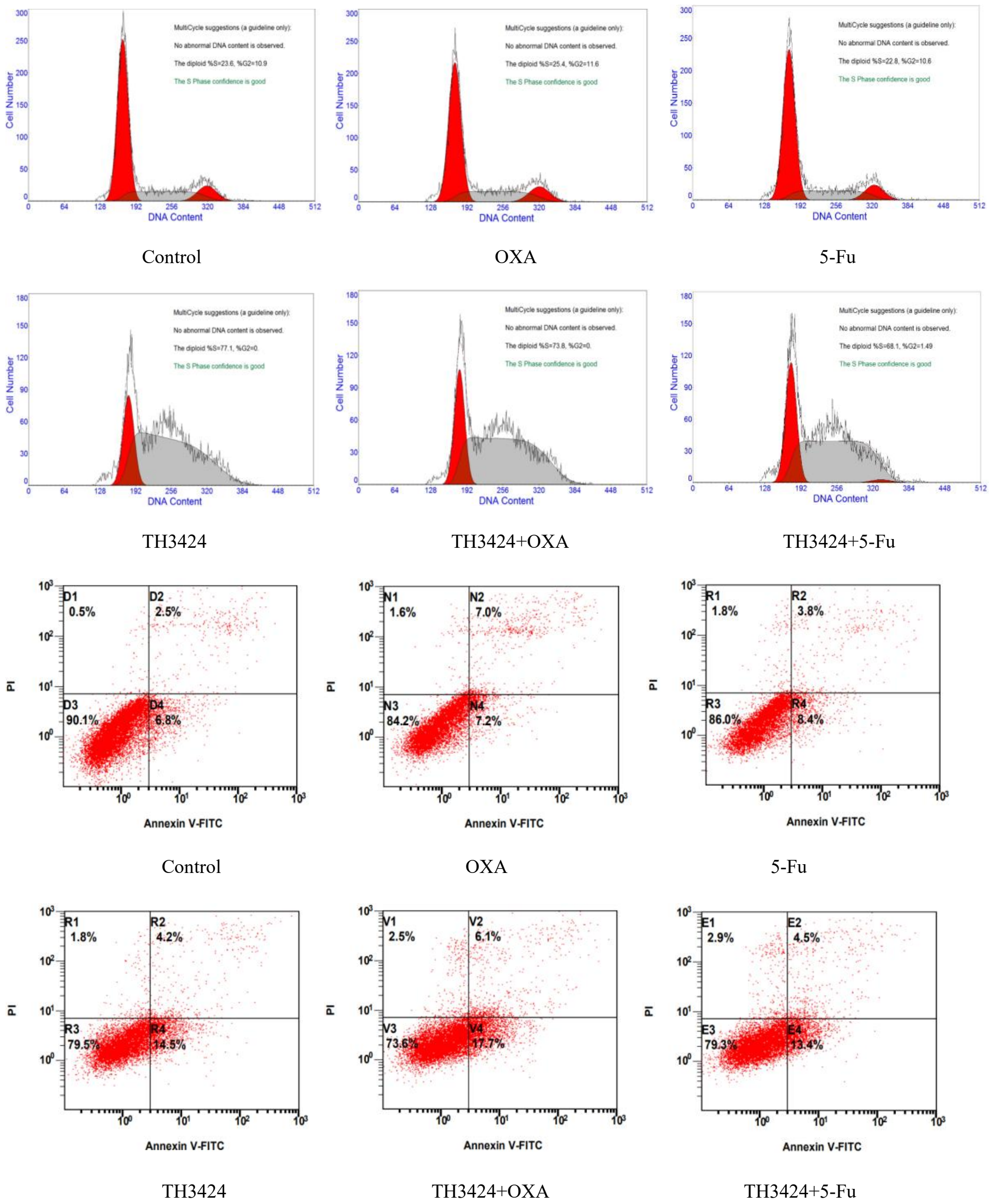

(a) DNA content-based cell cycle of HepG2 cells after TH3424(0.05μM), OXA(0.02μg/ml) and 5-Fu(0.3125μg/ml) alone or in combination treatment were analyzed using Flow cytometry.(b) Percentage of cells in S phases of HepG2 cells after TH3424(0.05μM), OXA(0.02μg/ml) and 5-Fu(0.3125μg/ml) alone or in combination treatment.

(c) Flow cytometry histograms of HepG2 cells after 48h treatment with TH3424(0.05μM), OXA(0.02μg/ml) and 5-Fu(0.3125μg/ml) alone or in combination, the samples were detected using AV/PI double-staining method.
